# Smart Solid‐State Interphases Enable High‐Safety and High‐Energy Practical Lithium Batteries

**DOI:** 10.1002/advs.202400600

**Published:** 2024-04-06

**Authors:** Yu Wu, Yuan Liu, Xuning Feng, Zhuang Ma, Xiaodong Xu, Dongsheng Ren, Xuebing Han, Yalun Li, Languang Lu, Li Wang, Xiangming He, Minggao Ouyang

**Affiliations:** ^1^ School of Materials Science and Engineering Beijing Institute of Technology Beijing 100081 China; ^2^ National Key Laboratory of Science and Technology on Materials under Shock and Impact Beijing Institute of Technology Beijing 100081 China; ^3^ State Key Laboratory of Intelligent Green Vehicle and Mobility Tsinghua University Beijing 100084 China; ^4^ Institute of Nuclear and New Energy Technology Tsinghua University Beijing 100084 China

**Keywords:** high‐energy, high‐safety, practical batteries, smart, solid‐state interphases

## Abstract

With the electrochemical performance of batteries approaching the bottleneck gradually, it is increasingly urgent to solve the safety issue. Herein, all‐in‐one strategy is ingeniously developed to design smart, safe, and simple (3S) practical pouch‐type LiNi_0.8_Co_0.1_Mn_0.1_O_2_||Graphite@SiO (NCM811||Gr@SiO) cell, taking full advantage of liquid and solid‐state electrolytes. Even under the harsh thermal abuse and high voltage condition (100 °C, 3–4.5 V), the pouch‐type 3S NCM811||Gr@SiO cell can present superior capacity retention of 84.6% after 250 cycles (based pouch cell: 47.8% after 250 cycles). More surprisingly, the designed 3S NCM811||Gr@SiO cell can efficiently improve self‐generated heat T_1_ by 45 °C, increase TR triggering temperature T_2_ by 40 °C, and decrease the TR highest T_3_ by 118 °C. These superior electrochemical and safety performances of practical 3S pouch‐type cells are attributed to the robust and stable anion‐induced electrode‐electrolyte interphases and local solid‐state electrolyte protection layer. All the fundamental findings break the conventional battery design guidelines and open up a new direction to develop practical high‐performance batteries.

## Graphical abstract

With the electrochemical performance of batteries approaching the bottleneck gradually, it is increasingly urgent to solve the safety issue. Herein, all‐in‐one strategy is ingeniously developed to design smart, safe, and simple practical pouch‐type cells, taking full advantage of liquid and solid‐state electrolytes. All the fundamental findings open up a new direction to develop high‐performance batteries.

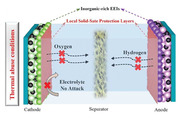

## 1 Introduction

In order to limit the effect of energy crisis and environmental pollution, great efforts have driven rapid progress in the fields of electric vehicles and large‐scale grid storage.^[^ ^1^, ^2^ ^]^ Lithium‐ion batteries (LIBs) are widely recognized for overall performance, rendering efficient energy storage and utilization.^[^ ^3^, ^4^, ^5^ ^]^ However, the issues of poor safety and specific energy hinder further promotion.^[^ ^6^, ^7^, ^8^ ^]^ The LIBs with higher safety and energy are urgently demanded, which will strongly enhance consumer's confidence.

To further enhance the energy density, developing high‐voltage batteries with high‐capacity electrode is of critical importance. Among numerous candidates, LiNi_0.8_Co_0.1_Mn_0.1_O_2_ (NCM811) oxides with high capacity are the most promising cathodes for practical high‐energy LIBs,^[^ ^9^, ^10^, ^11^, ^12^ ^]^ meanwhile the mixtures of SiO and graphite are becoming increasingly attractive in anode choices.^[^ ^13^, ^14^, ^15^ ^]^ Attributed to its highest energy density in current commercial LIBs, LiNi_0.8_Co_0.1_Mn_0.1_O_2_||Graphite@SiO (NCM811||Gr@SiO) cell has received tremendous attention in both academic and industrial research.^[^ ^16^ ^]^ However, the phase transition of the NCM811 cathode and huge volume change of alloy anode led to inferior electrochemical performances and serious security risks characterized by thermal runaway (TR), especially at high operation potential.^[^ ^17^, ^18^, ^19^ ^]^

Tightly connected to the electrode material like “blood”, the electrolyte is a key factor in the optimal performance of the LIBs.^[^ ^20^, ^21^, ^22^ ^]^ However, the traditional ethylene carbonate (EC)‐based electrolyte only has limited oxidation stability (≈4.3 V), resulting in the inability to operate at high potential.^[^ ^23^, ^24^, ^25^ ^]^ In addition, the interphase formed on Gr@SiO anode cycled in traditional electrolyte is unstable. Moreover, the severe exothermic reaction between EC solvent and NCM811 triggers the TR.^[^ ^26^, ^27^, ^28^ ^]^ The traditional EC‐based electrolyte has been employed since the commercialization of LIBs, but it is far from enough to meet the requirements of next‐generation LIBs ^[^ ^29^ ^]^ Designing EC‐free electrolytes is the most promising approach because it solves the security risks and limited oxidation stability issues from the root. However, the current EC‐free electrolyte cannot be immediately useful in commercial leading NCM811||Gr@SiO cell due to the gas evolution, expansion, and security issues from crosstalk. Obviously, the development of EC‐free electrolytes for practical high‐energy NCM811||Gr@SiO cells is needed. In addition, solid‐state electrolytes are regarded as good choices to further improve the safety performance.^[^ ^30^, ^31^, ^32^ ^]^ Nevertheless, the low ionic conductivity and insufficient interfacial contact lead to poor electrochemical performances.^[^ ^33^, ^34^ ^]^ Taking full advantage of the superior properties of liquid and solid‐state electrolytes, the ingenious design strategy is urgently required to develop the next generation advanced LIBs.

**Figure 1:**
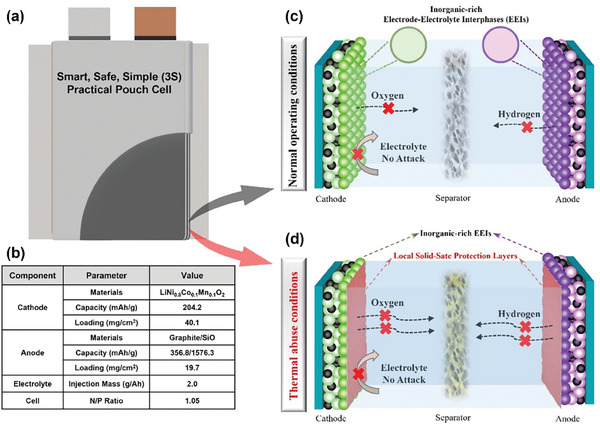
The design diagram of a smart, safe, and simple (3S) cell. a,b) The structure and specific parameters of the practical pouch‐type cell, respectively. c,d) The operation diagram of 3S pouch‐type cells under normal and thermal abuse conditions, respectively.

In this work, we propose a smart high‐safety and high‐energy practical battery via all‐in‐one in situ local polymerization strategy, which fully combines the advantages of liquid and solid‐state electrolytes (**Figure** **1**). In detail, under normal operating conditions, the liquid EC‐free electrolyte can efficiently passivate the highly catalytic active NCM811 cathode surface and form a high‐quality interphase to obtain good compatibility with the Gr@SiO anode. When the battery temperature rises to 100 °C due to abuse, the temperature‐responsive additive polymerizes rapidly to intelligently form a local solid‐state electrolyte protection layer, which can greatly improve safety by preventing internal short circuit, cutting off the exothermic reaction triggered by direct contact between electrode and electrolyte, and inhibiting the crosstalk reaction caused by oxygen release from NCM811 cathode and hydrogen production from Gr@SiO anode. In detail, the designed NCM811||Gr@SiO pouch cell can efficiently improve self‐generated heat T_1_ by 45 °C, increase TR triggering temperature T_2_ by 40 °C, and decrease the TR highest T_3_ by 118 °C. More surprisingly, the NCM811||Gr@SiO pouch cell presents superior capacity retention of 84.6% after 250 cycles (based pouch cell: 47.8% after 250 cycles) and increased initial capacity (21.0%) under the harsh thermal abuse and high voltage condition (100 °C, 3—4.5 V). The work sheds light on a new direction for designing the smart, safe, and simple electrolyte to develop practical high‐performance batteries.

## 2 Results and Discussion

To solve the inherent disadvantages of thermodynamic limitations and poor thermal stability with electrode for conventional EC‐based electrolyte, EC solvent was replaced and LiFSI‐LiPF_6_ dual‐salt was introduced to improve the ionic conductivity, interfacial compatibility, oxidation resistance, and stability with electrode. Then, the designed electrolyte system (0.6 m LiPF_6_‐0.9 m LiFSI/EMC) was simply mixed with poly(ethylene glycol) methyl ether methacrylate and 2,2,3,3,3‐pentafluoropropyl acryla (at molar ratio of 2:1) to achieve smart temperature response in situ interface local polymerization for high safety practical pouch cell, which is defined as smart, safe, and simple (“3S”) via the ingenious all‐in‐one design. As shown in Figure S1 (Supporting Information), the liquid smart functional additive (Figure S1a,b, Supporting Information) was successfully transformed into solid state (Figure S1c,d, Supporting Information) after suffering from thermal abuse, which was heated from 25 to 100 °C. Under normal operating conditions, the liquid 3S EC‐free electrolyte can efficiently passivate the NCM811 and the Gr@SiO anode by forming stable inorganic‐rich electrode‐electrolyte interphases (EEIs), which guarantees excellent electrochemical performance and high‐safety runaway threshold (Figure S1e–h, Supporting Information). Moreover, when the cell temperature rises to 100 °C due to thermal abuse, the 3S EC‐free electrolyte polymerizes rapidly to intelligently form a local solid‐state electrolyte protection layer (Figure S1i–l, Supporting Information), which can greatly improve safety and high voltage electrochemical performance.

**Figure 2:**
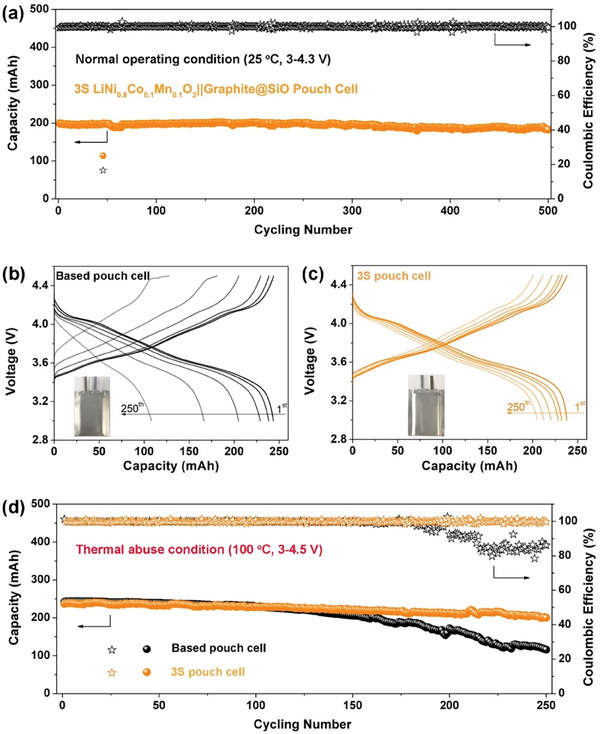
Electrochemical behavior of NCM811||Gr@SiO pouch cells. a) Cycling stability of 3S NCM811||Gr@SiO pouch cell charged up to 4.3 V under normal conditions. b,c) Charge–discharge profiles of based and 3S NCM811||Gr@SiO pouch cell charged up to 4.5 V, respectively. d) Cycling performances of based and 3S NCM811||Gr@SiO pouch cell charged up to 4.5 V after 100 °C thermal abuse.

The cycling properties of practical NCM811||Gr@SiO pouch cells with different electrolyte systems are exhibited in **Figure** **2**. With the combination of EC‐free electrolyte and functional additives, the 3S NCM811||Gr@SiO pouch cell was successfully designed. First, the 3S NCM811||Gr@SiO pouch cell presented superior long‐term capacity retention of 93.3% and steady average coulombic efficiency (CE) of >99.94% after 500 cycles under normal conditions (Figure 2a). Under harsh thermal abuse and high voltage conditions (100 °C, 3–4.5 V), the cycling performances were further investigated. By enhancing the operation voltage from 4.3 to 4.5 V, the discharge capacity of 3S NCM811||Gr@SiO pouch cell can be increased from 198.9 to 240.6 mAh. Notably, the discharge capacity of the 3S NCM811||Gr@SiO cell can be improved by 21.0%. Moreover, the 3S NCM811||Gr@SiO pouch cell presents more stable voltage profiles compared to the based pouch cell (Figure 2b,c), attributed to the reduced side reactions during the cycling after thermal abuse. Specially, the based pouch cell undergoes a sustained capacity fading from 100 cycles (47.8% after 250 cycles). In addition, the CE of based pouch cell drops dramatically from 175 cycles. In sharp contrast, the 3S NCM811||Gr@SiO pouch cell presents stable capacity retention of 84.6% and average CE of 99.88% after 250 cycles (Figure 2d). These superior cycling performances prove that the formation of stable EEIs effectively suppresses parasitic reactions. More importantly, the construction of an in situ local solid‐state electrolyte protection layer after thermal abuse can further ensure the efficient operation of the practical pouch cell in the extreme conditions of high temperature and voltage.

**Figure 3:**
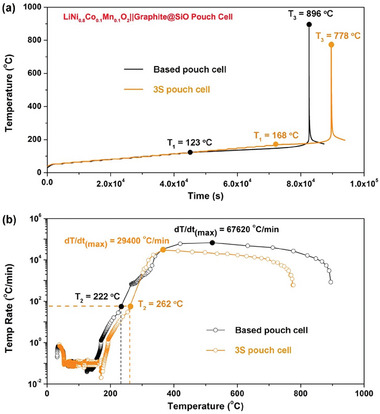
Thermal runaway characteristics of practical pouch‐type NCM811||Gr@SiO cells. a) The heat‐wait‐search curves of based and 3S NCM811||Gr@SiO pouch cells. b) Temperature rate curves of based and 3S NCM811||Gr@SiO pouch cells under ARC test.

The TR characteristics of NCM811||Gr@SiO pouch cells were investigated by accelerating rate calorimetry (ARC), which can effectively define three key temperatures {T_1_, T_2_, T_3_}.^[^ ^35^, ^36^, ^37^, ^38^ ^]^ Increasing T_1_, maximizing T_2_, and minimizing T_3_ are critical to improving safety of LIBs.^[^ ^39^ ^]^ The Ah‐level NCM811||Gr@SiO pouch cell employing based electrolyte yields the self‐generated heat T_1_ of 123 °C, then the TR is triggered at 222 °C (T_2_) (**Figure** **3**a,b). Surprisingly, the T_1_ and T_2_ temperature of the 3S NCM811||Gr@SiO pouch cell can be enhanced up to 168 and 262 °C (Figure 3a,b), respectively. It is worth noting that the self‐generated heat temperature has increased from 123 to 168 °C, attributed to the synergistic effect of stable anion‐induced inorganic‐rich EEIs and the local solid‐state electrolyte protection layer. Moreover, the TR highest temperature T_3_ can be reduced by 118 °C. In summary, Ah‐level 3S NCM811||Gr@SiO pouch cell can efficiently improve self‐generated heat T_1_ by 45 °C, improve TR triggering temperature T_2_ by 40 °C, and decrease the TR highest T_3_ by 118 °C, indicating that the cell has a very high thermal runaway threshold. To the best of our knowledge, this is the best safety performance ever reported.^[^ ^16^, ^26^, ^28^, ^38^, ^40^, ^41^ ^]^ In addition to employing ARC to accurately capture the internal temperature of the practical cells, lateral heating test is also conducted to further evaluate the TR temperature of the outer surface of the cells. The practical‐based pouch cell shows the TR trigger temperature (T_trigger_) and TR maximum temperature (T_max_) of 121.1 and 513.1 °C, respectively (Figure S2a, Supporting Information). In sharp contrast, the 3S NCM811||Gr@SiO cell achieves higher T_trigger_ of 153.1^ o^C and lower T_max_ 445.4 °C, respectively (Figure S2b, Supporting Information). Attributed to the robust and stable anion‐induced EEIs and local solid‐state electrolyte protection layer, the intrinsic safety of practical 3S NCM811||Gr@SiO pouch cell can be greatly enhanced.

**Figure 4:**
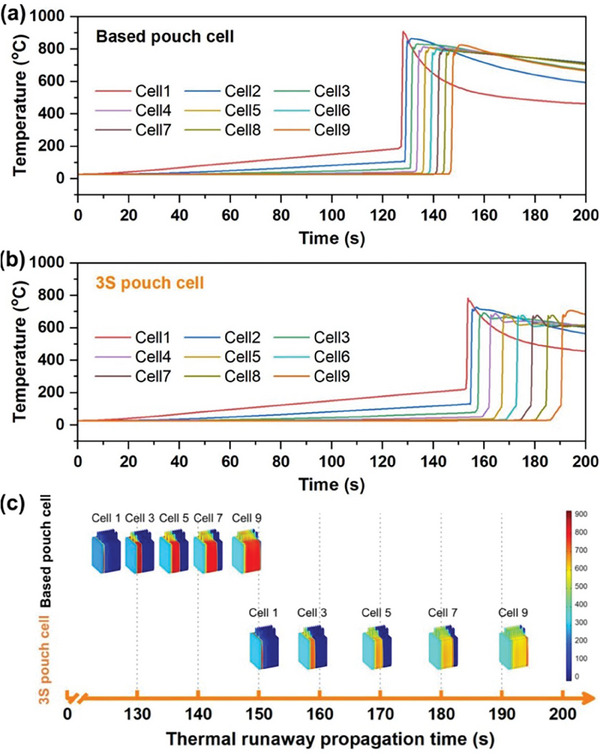
a,b) The temperature curves of based and 3S 9‐cell modules calculated in the TR propagation simulation, respectively. c) The propagation temperature‐time sequence map of the 3D module model.

In addition to the safety features of cell level, the TR propagation characteristics of the module level are extremely important in practical applications.^[^ ^39^ ^]^ As shown in **Figure** **4**, COMSOL Multiphysics was employed to establish ED models and present the temperature distributions of the heat‐induced TR propagation for 9‐cell module using different electrolytes. The average propagation time of the based pouch cell yields 2.5 s from cell x to x+1 (Figure 4a). However, the 3S pouch cell can effectively extend the propagation time to 4.8 s (Figure 4b), which can increase by 92%. More surprisingly, while all nine based pouch cells are triggered, the first 3S pouch cell is still safe (Figure 4c). Significant suppression of TR propagation within module because of the improved safety of the cell with the robust and stable anion‐induced EEIs and local solid‐state electrolyte protection layer.

**Figure 5:**
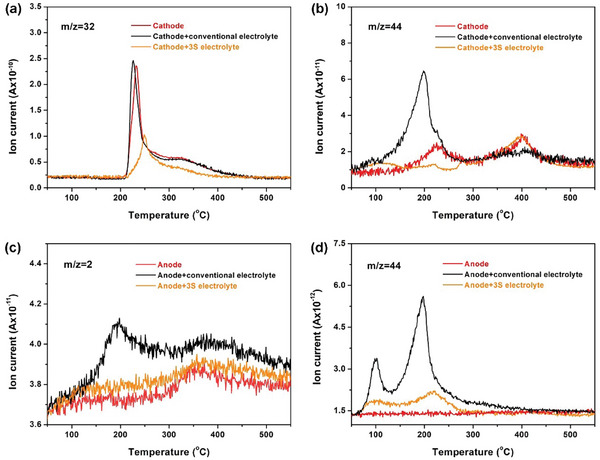
a,b) The curves of oxygen (m/z = 32) and carbon dioxide (m/z = 44) released from charged NCM811 cathode with different electrolyte, respectively. c,d) The curves of hydrogen (m/z = 2) and carbon dioxide (m/z = 44) released from charged Gr@SiO anode with different electrolyte, respectively.

The TR characteristics of NCM811||Gr@SiO cell is closely related to the chain crosstalk reaction triggered by charged electrode with electrolyte.^[^ ^42^, ^43^ ^]^ In this regard, the differential scanning calorimeter coupled with mass spectrometry (DSC‐MS) test was employed to study the crosstalk between the electrode and electrolyte. **Figure** **5**a shows the curves of the oxygen (m/z = 32) released from charged NCM811 cathode with different electrolytes. Compared with the pure NCM811 cathode, the release of oxygen after adding based electrolyte will be more violent. Interestingly, the cathode with 3S electrolyte shows a “halving” oxygen release intensity and a greatly reduced rate. A similar trend appears on the carbon dioxide (m/z = 44) curves (Figure 5b). When replaced by 3S electrolyte, the carbon dioxide produced by the charged NCM811 cathode with electrolyte is basically completely suppressed. In addition to the crosstalk between the cathode and the electrolyte represented by oxygen, the crosstalk between the charged anode and electrolyte, represented by hydrogen, is responsible for the thermal failure. As shown in Figure 5c, the release of hydrogen (m/z = 2) increases rapidly from ≈150 °C for the fully charged Gr@SiO anode with conventional electrolyte. In sharp contrast, the release of hydrogen of 3S electrolyte can be markedly suppressed. Moreover, compared with the violent release of carbon dioxide (m/z = 44) of Gr@SiO anode with conventional electrolyte, the 3S electrolyte exhibits the greatly weakened intensity and rate (Figure 5d). The above‐mentioned results above prove that TR crosstalk reactions at the material level can be significantly inhibited in the 3S electrolyte, which is consistent with the superior safety properties of 3S pouch‐type NCM811||Gr@SiO cells.

**Figure 6:**
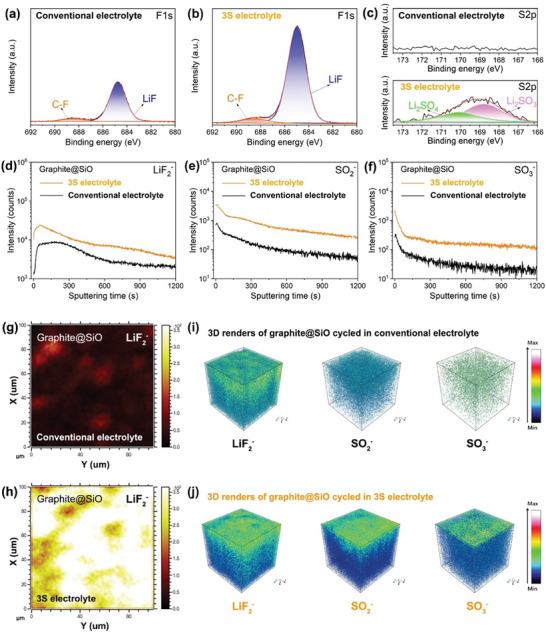
a–c) XPS spectra of F1s and S2p for the SEI of Gr@SiO anode cycled in conventional electrolyte and 3S electrolyte. d–f) ToF‐SIMS spectra of the Gr@SiO cycled in different electrolytes. g,h) X‐Y plane chemical maps of LiF_2_^−^ species of the SEI formed on Gr@SiO with different electrolyte. i,j) 3D renders of LiF_2_^−^, SO_2_^−^, and SO_3_^−^ species of the SEI of Gr@SiO cycled in conventional and 3S electrolyte, respectively.

The formation of EEIs to stabilize electrodes plays a vital role on the safety and electrochemical characteristics of LIBs. As shown in **Figure** **6**, the XPS and ToF‐SIMS technologies were employed to disclose the chemistry and structure of the solid‐electrolyte interphase (SEI). The F1s spectra of Gr@SiO anode clearly reveal that SEI formed by the 3S electrolyte contains more LiF species, evidenced by the higher intensity of LiF peak at 684.8 eV (Figure 6a,b).^[^ ^44^ ^]^ Moreover, the S2p spectra are conducted to further confirm the chemical compositions. The S species are only observed on SEI of the graphite employing 3S electrolyte, derived from the salt anions (Figure 6c). To further evaluate the SEI formed in different electrolytes, secondary‐ion fragments (e.g., LiF_2_^−^, SO_2_^−^, and SO_3_^−^) were detected by using ToF‐SIMS. As shown in Figure 6d–f, the strong signals of LiF_2_^−^, SO_2_^−^, and SO_3_^−^ species were detected on the interphase of Gr@SiO cycled in the 3S electrolyte, indicating the SEI contains more robust inorganic species.^[^ ^45^ ^]^ These results of secondary‐ion fragments are consistent with the above XPS results. Compared to the poor LiF_2_^−^ species X‐Y distribution (100*100 um) of Gr@SiO cycled in conventional electrolyte (Figure 6g), the interphase of Gr@SiO cycled in 3S electrolyte presented the abundant inorganic LiF_2_^−^ species (Figure 6h). Furthermore, the 3D‐rendering images visually show that the SEI of Gr@SiO cycled in 3S electrolyte contains a richer and more homogeneous distribution of inorganic LiF_2_^−^, SO_2_^−^, and SO_3_^−^ species compared to the conventional electrolyte (Figure 6i,j). The anions‐induced stable inorganic‐rich SEI is beneficial to suppressing parasitic reactions and supporting reversible Li^+^‐intercalation behavior (Figure 2a,d). In addition, the more inorganic components of the SEI with higher thermal stability effectively reduce the self‐exothermic heat and crosstalk hydrogen associated with the Gr@SiO anode of the pouch cell.

**Figure 7:**
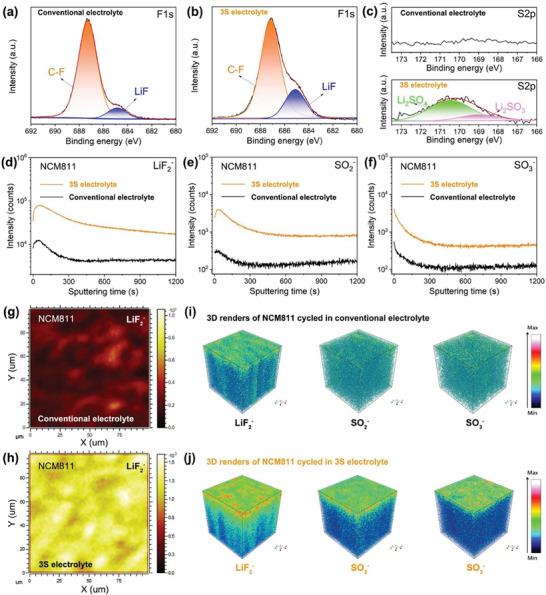
a–c) XPS spectra of F1s and S2p for the CEI of NCM811 cathode cycled in conventional electrolyte and 3S electrolyte. d–f) ToF‐SIMS spectra of the NCM811 cycled in a different electrolyte. g,h) X‐Y plane chemical maps of LiF_2_^−^ species of the CEI formed on NCM811 with different electrolyte. i,j) 3D renders of LiF_2_^−^, SO_2_^−^, and SO_3_^−^ species of the CEI of NCM811 cycled in conventional and 3S electrolyte, respectively.

In addition to passivating the Gr@SiO, the designed 3S electrolyte also effectively protects the NCM811. Similar to the chemistry of interphase formed on the Gr@SiO anode, the interphase of NCM811 cathode cycled in 3S electrolyte contains more inorganic components. The XPS was first adopted to evaluate the chemical compositions of the cathode‐electrolyte interphase (CEI). As shown in **Figure** **7**a,b, a higher LiF peak (684.8 eV) can be obtained in the NCM811 with 3S electrolyte. Moreover, S2p peak is only observed on CEI for the NCM811 employing 3S electrolyte, attributed to the decomposition of salt anions (Figure 7c).^[^ ^46^ ^]^ Plenty of fragments (e.g., LiF_2_^−^, SO_2_^−^, and SO_3_^−^) were obtained by using ToF‐SIMS to reveal the depth distribution of the CEI (Figure 7d–f). Compared to the uneven and poor inorganic species (LiF_2_^−^, SO_2_^−^, and SO_3_^−^) distribution of NCM811 cycled in conventional electrolyte, the interphase of NCM811 cycled in 3S electrolyte presented the more uniform and richer 2D (Figure 7g,h) and 3D (Figure 7i,j) distribution of inorganic species, attributed to the decomposition of salt anions. Combining the high‐voltage cyclability performances, the robust and stable inorganic‐rich CEI is beneficial to suppress the evolution of lattice oxygen resulted from phase transition, and prevent from the parasitic reactions between highly reactive NCM811 and the electrolyte.

## 3 Conclusion

By taking full advantage of liquid and solid‐state electrolytes, we have ingeniously developed a smart, safe, and simple (3S) practical pouch‐type NCM811||Gr@SiO cell via all‐in‐one strategy. In detail, EC solvent was replaced and LiFSI‐LiPF_6_ dual‐salt was introduced to improve the ionic conductivity, interfacial compatibility, oxidation resistance, and stability with electrode. Then, the EC‐free electrolyte was simply mixed with ester functional additives to achieve smart temperature response in situ interface local polymerization. Attributed to the robust and stable anion‐induced EEIs and local solid‐state electrolyte protection layer derived from 3S EC‐free electrolyte, the electrochemical and safety properties of practical NCM811||Gr@SiO pouch cell can be greatly enhanced. The 3S NCM811||Gr@SiO pouch cell exhibited excellent capacity retention of 93.3% after 500 cycles under normal conditions. Under harsh thermal abuse and high voltage condition (100 °C, 3–4.5 V), the 3S NCM811||Gr@SiO pouch cell presents superior capacity retention of 84.6% after 250 cycles (based pouch cell: 47.8% after 250 cycles) and increased initial capacity (21.0%). More surprisingly, the designed 3S NCM811||Gr@SiO pouch cell can efficiently improve self‐generated heat T_1_ by 45 °C, increase TR triggering temperature T_2_ by 40 °C, and decrease the TR highest T_3_ by 118 °C. All these fundamental findings break the conventional battery design guidelines and extend the conventional knowledge of liquid‐solid state electrolyte systems. The work sheds light on a new direction for designing the smart, safe, and simple electrolyte to develop practical high‐performance batteries.

## Conflict of Interest

The authors declare no conflict of interest.

## Supporting information

Supporting Information

## Data Availability

The data that support the findings of this study are available from the corresponding author upon reasonable request.
