# Supplementary material for: Smart Solid‐State Interphases Enable High‐Safety and High‐Energy Practical Lithium Batteries
Source: Adv Sci (Weinh). 2024 Apr 6;11(22):2400600. doi: 10.1002/advs.202400600 (PMC11165460; doi:10.1002/advs.202400600)
Supplement: Supplementary file 1 — Supporting Information [file ADVS-11-2400600-s001.pdf]

ADVANCED SCIENCE
Open Access

Supporting Information

for *Adv. Sci.*, DOI 10.1002/advs.202400600

Smart Solid-State Interphases Enable High-Safety and High-Energy Practical Lithium Batteries

Yu Wu, Yuan Liu, Xuning Feng*, Zhuang Ma, Xiaodong Xu, Dongsheng Ren, Xuebing Han, Yalun Li, Languang Lu, Li Wang, Xiangming He and Minggao Ouyang**

Supporting Information

Smart Solid-State Interphases enable High-Safety and High-Energy Practical Lithium Batteries

Yu Wu,[1,2,*] Yuan Liu,[1] Xuning Feng,[3,*] Zhuang Ma,[1,2] Xiaodong Xu,[3] Dongsheng Ren,[3] Xuebing Han,[3] Yalun Li,[3] Languang Lu,[3] Li Wang,[4] Xiangming He,[4] Minggao Ouyang[3,*]

[1] School of Materials Science and Engineering, Beijing Institute of Technology, Beijing 100081, China.
[2] National Key Laboratory of Science and Technology on Materials under Shock and Impact, Beijing Institute of Technology, Beijing 100081, China.
[3] State Key Laboratory of Intelligent Green Vehicle and Mobility, Tsinghua University, Beijing 100084, China.
[4] Institute of Nuclear and New Energy Technology, Tsinghua University, Beijing 100084, China.

Corresponding authors: wuyu@bit.edu.cn; fxn17@mail.tsinghua.edu.cn; ouymg@mail.tsinghua.edu.cn

Materials. Machine-made Ah-level NCM811||Gr@SiO pouch-type cells were obtained dry (no electrolyte) from LiFun Technology (Xinma Industry Zone). The cells were transferred to an argon-filled glove box without exposure to ambient air, where they were filled with lean electrolyte (typical industry level: 2 mL electrolyte was injected into the 1 Ah pouch-type cells), and the formation of the cells was performed at C/10 under 40 ^{o}C. In this work, the based electrolyte was 1 M $LiPF_6$ in EC/EMC. The poly(ethylene glycol) methyl ether meth-acrylate and 2,2,3,3,3-pentafluoropropyl acryla were obtained from SIGMA-ALDRICH and dried over activated molecular sieves before use.

Safety tests. An EV-ARC manufactured by Thermal Hazard Technology was utilized for thermal runaway (TR) tests. A K type thermocouple was inserted into the center of cell to measure the internal temperature, which is used to evaluate the cell TR performance. During the TR tests, the EV-ARC was operated under the heat-wait-seek mode. A heating step of 5 ^{o}C with a wait time of 15 min was performed on the ARC starting from 40 ^{o}C. The EV-ARC system would go into the exotherm mode to track the temperature rise of tested batteries and maintain the adiabatic condition if the measured temperature rate exceeded 0.01 $^{o}C\cdot min^{-1}$. The lateral heating test can be used to determine the cell's thermal stability following a failure event. The ceramic heating plate measured 50 mm in length, 50 mm in width, and 2 mm in thickness. In this experiment, the cell was fully charged and a Teflon tape was used to securely attach the cell sample to the heating sheet. Voltage lines and thermocouples were used to record the voltage and temperature of the cell.

Characterizations. The differential scanning calorimeter coupled with mass spectrometry (DSC-MS) test, which is performed on a Netzsch STA449-QMS, can be used to determine the thermal stability of the electrode materials. ToF-SIMS measurements were performed to probe the depth distribution on the surface of the Gr@SiO anode and NCM811 cathode (ION-TOF TOF-SIMS 5, Germany). X-ray photoelectron spectroscopy (XPS) (PHI Quanteral II, Japan) was conducted to evaluate the chemical composition in the prepared Gr@SiO anode and NCM811 cathode.

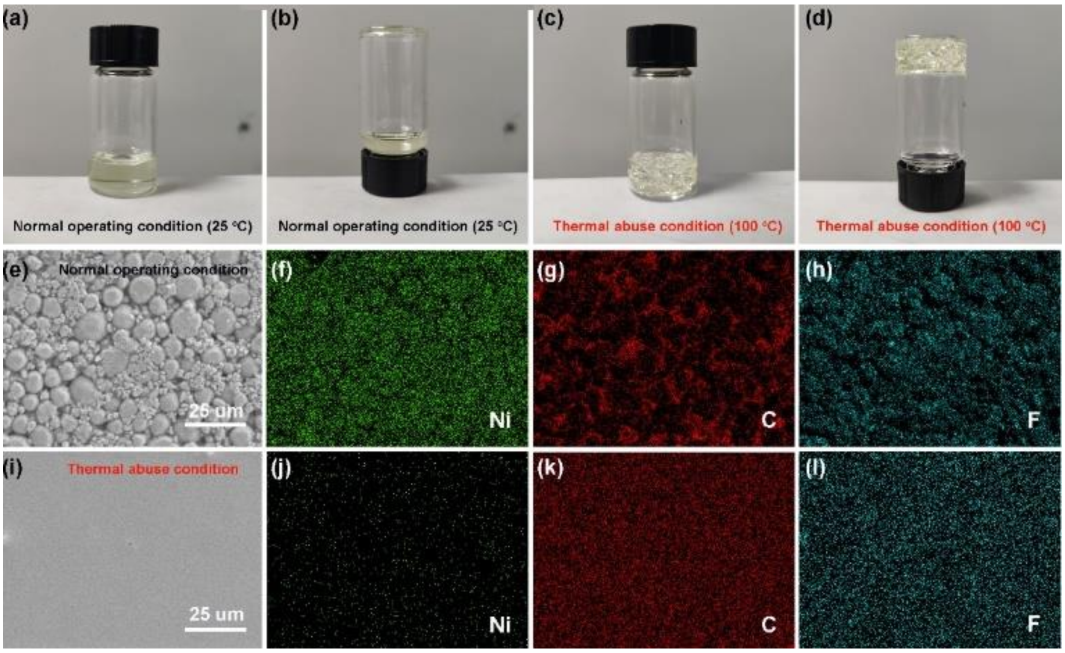

Figure S1. a-d) Optical photographs of functional additive under 25 ^{o}C and 100 ^{o}C, respectively. e-h) The surface SEM and EDS mapping of NCM811 electrode with 3S EC-free electrolyte under normal operating condition. i-l) The surface SEM and EDS mapping of NCM811 electrode with 3S EC-free electrolyte under thermal abuse conditions.

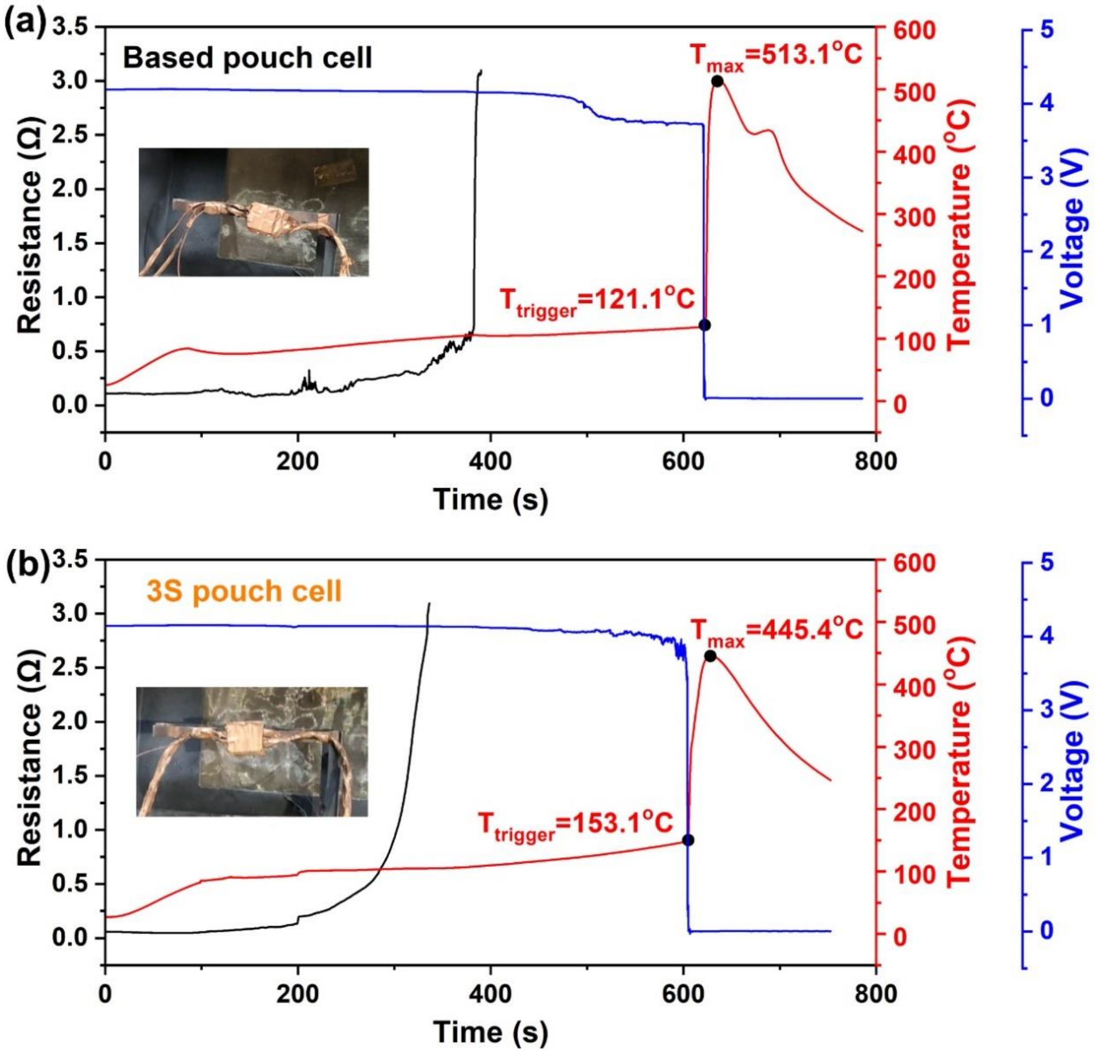

Figure S2. Safety features of practical NCM811||Gr@SiO pouch cells. a) The plots of charged based NCM811||Gr@SiO pouch cell under lateral heating test. b) The plots of charged 3S NCM811||Gr@SiO pouch cell under lateral heating test.
